# Oral phenotype in SATB2-associated syndrome: cross-sectional study of the French cohort

**DOI:** 10.1186/s13023-025-03609-3

**Published:** 2025-06-05

**Authors:** Nancy Vegas, Marlène Rio, Pauline Adnot, Véronique Soupre, Florence Petit, Jamal Ghoumid, Annick Toutain, Klaus Dieterich, Isabelle Marey, Brigitte Gilbert-Dussardier, Gwenaël Le Guyader, Christine Francannet, Elise Schaefer, Laurence Perrin, Mathilde Nizon, Claire Beneteau, David Genevieve, Marjolaine Willems, Laurence Faivre, Marianne Grimaldi, Judith Melki, Radka Stoeva, Audrey Putoux, Linda Pons, Karelle Benistan, Jeanne Amiel, Véronique Abadie

**Affiliations:** 1 General Pediatrics Unit, Necker University Hospital, Assistance Publique-Hôpitaux de Paris, 149 rue de Sèvres, 75015 Paris, France; 2 Malformation and Embryology Laboratory, IMAGINE Institute, Paris, France; 3 Université Paris Cité, Paris, France; 4 Reference Centre for Rare Diseases Centre de Référence Maladies Rares « Syndromes de Pierre Robin et troubles de succion-déglutition congénitaux », Necker University Hospital, Paris, France; 5 Medical Genetics Department, Necker University Hospital, Paris, France; 6 Maxillo-Facial and Plastic Surgery Unit, Necker University Hospital, Paris, France; 7 Clinique de Génétique Guy Fontaine, CHU Lille, Hôpital Jeanne de Flandre, Lille, France; 8 Service de Génétique, CHRU de Tours, Hôpital Bretonneau, Tours, France; 9 Unité de Génétique Clinique, CHU Grenoble, Hôpital Couple-Enfant, Grenoble, France; 10 Service de Génétique Médicale, CHU de Poitiers, Poitiers, France; 11 Service de Génétique Médicale, CHU de Clermont-Ferrand, Hôpital d’Estaing, Clermont-Ferrand, France; 12 Service de Génétique Médicale, CHU de Strasbourg, Hôpital de Hautepierre, Strasbourg, France; 13 Unité de Génétique Clinique, CHU Paris, Hôpital Robert Debré, Assistance Publique-Hôpitaux de Paris, Paris, France; 14 Unité de Génétique Clinique, CHU de Nantes, Nantes, France; 15 Département de Génétique Médicale, CHU de Montpellier, Hôpital Arnaud de Villeneuve, Montpellier, France; 16 Centre de Génétique, Hôpital d’enfants, CHU Dijon Bourgogne, Hôpital François Mitterand, Dijon, France; 17 Unité de Génétique Médicale – Médecine néonatale, Centre Hospitalier Sud Francilien and INSERM, UMR-1195, Le Kremlin-Bicêtre, France; 18 Laboratoire de Génétique Médicale et Cytogénétique, Centre Hospitalier Le Mans, Le Mans, France; 19 Service de Génétique, CHU de Lyon, GH Est-Hôpital Femme Mère Enfant, Lyon, France; 20 Laboratoire de Biologie Médicale, Unité Fonctionnelle de Cytogénétique, Centre Hospitalier de Valence, Valence, France; 21 Service de Génétique, Hôpital Raymond Poincaré, Assistance Publique-Hôpitaux de Paris, Garches, France

**Keywords:** SATB2 syndrome, Speech delay, Psychomotor delay, Robin sequence

## Abstract

**Background:**

SATB2-associated syndrome (SAS) results from various mutations of the *SATB2* gene and associates a neurodevelopmental disorder including major speech delay, intellectual disability, and behavioral problems with dental anomalies, sometimes a cleft palate, risk of osteoporosis, and facial dysmorphism. The principal objective of this study was to describe the oral phenotype of young children with SATB2-associated syndrome, especially in terms of orofacial malformation of Robin Sequence (RS) spectrum (bifid uvula, cleft palate, or RS, dental malformation, feeding and communication, with data from a national cohort. The secondary objective was to determine whether feeding and communication disorders were more severe when associated with an orofacial malformation of RS spectrum.

**Methods:**

We conducted a retrospective cross-sectional study among the largest possible cohort of patients with a mutation of the *SATB2* gene in France. A questionnaire completed by the referring physicians and by telephone with parents enabled us to collect the following clinical information: (1) orofacial morphology, feeding difficulties, and pharyngeal functioning from birth to 3 years, (2) communication and language from 0 to 6 years, (3) speech development at the last examination.

**Results:**

The study included 40 patients. Early and persistent feeding difficulties were found in 55% of the children. Communication was abnormal from the first months of life, with poor babbling in 85% of them. A major language delay was described in all patients; 65% had a vocabulary of 10 words or less. An anomaly of RS spectrum was found in half the cases, and dental malformations were described in 90%. Feeding difficulties and language delay were greater in the group with one or more orofacial malformations than the group with none.

**Conclusion:**

This study confirmed the severity of oral involvement, affecting feeding and speech simultaneously, in individuals with SAS. It raises the question of why the oral phenotype involving feeding and speech is more severe in the presence of cleft palate or RS. We recommend close monitoring of prelanguage communication in infants with apparently isolated cleft palate or RS and the search for *SATB2* impairment when a cleft palate or RS is found, especially in the prenatal period.

## Introduction

SATB2-associated syndrome (SAS), associated with abnormalities of the *SATB2* gene (OMIM 608148), is an entity described in 2014 by Docker et al. [1] after the observation that children born with a deletion in the 2q33.1 region have a clinical phenotype similar to that of children with a point mutation in the *SATB2* gene. The story began more than 30 years ago when Glass et al. [2] described a 16-year-old male with major intellectual disability, absent speech, dysmorphism, and areas of cutaneous depigmentation, all associated with deletion of the long arm of chromosome 2 at 2q32.2-2q33.1 (Glass syndrome). Several teams later described children with deletions of variable size in the 2q32-33 region but always containing the *SATB2* gene [3]. The similarities of the clinical manifestations of children with isolated *SATB2* mutations and those with deletions containing this gene demonstrate its involvement in the phenotype [4–7]. SAS clinical phenotype combines intellectual disability, including severe speech delay, especially marked for expressive speech, and behavioral problems (autism spectrum disorder, agitation, sleep disorders) with dental anomalies, cleft palates, and facial dysmorphism [4, 6, 8, 9]. More recently bone fragility has been described with a risk of fractures from the age of 10 years [10–12].

As the French national reference team for children born with a Robin sequence (RS), we became concerned several years ago when we observed unexpected poor later development in communication, speech, language, and cognition of children under management at birth for apparently isolated RS; that is, at birth, these children had RS (retrognathia—glossoptosis—posterior cleft palate (CP)—respiratory obstruction) but no additional organ malformation and neither any recognizable morphologic anomaly in the first months of life nor any anomaly in neonatal neurological examination. Several of them had SAS.

The involvement of SATB2 in CP dates back 2 decades. In 1999, Brewer et al. [13] described two young girls with posterior CP associated with moderate intellectual disability and carrying a cytogenetic anomaly involving the 2q32 locus. This locus was then identified as potentially responsible for isolated CP. The association between this locus involved in CP and the *SATB2* gene was established 4 years later by Fitzpatrick et al. [14]. Hence, *SATB2* is now one of the genes for which haploinsufficiency is known to be a potential cause of CP [15]. Similarly, associations between the *SATB2* gene and RS were first noted in 2001 by the description of an infant with RS in whom an interstitial 2q32-33 deletion was identified [16]. *SATB2* was not mentioned in this older report, but since then, it has become one of the genes potentially involved in syndromic RS [17].

The mechanism by which the haploinsufficiency of the *SATB2* gene induces RS is unclear. The *SATB2* gene codes for a protein in the family of special AT-rich sequence-binding protein-2 (SATB2), a factor regulating transcription and remodeling of chromatin [18, 19]. *SATB2* is expressed during embryogenesis in the craniofacial region, where it regulates osteoblast differentiation and craniofacial morphogenesis [20]. In mice, *Satb2* plays a role in the morphogenesis of the skeleton by its activity regulating functional osteoblastic genes, such as *Runx2*, *Atf4,* and *Sox9* [21]. The role of *Sox9* in RS is known by its direct involvement in campomelic dwarfism (severe osteodystrophy—RS—sexual ambiguity) [22] and by the involvement of mutations in regulatory regions upstream of *Sox9* in familial forms of isolated RS [23, 24].

The first objective of this national cross-sectional retrospective study was to describe the oral phenotype of children with SAS, namely their neonatal anatomy regarding CP, bifid uvula (BU) or Robin Sequence; neonatal respiratory and feeding disorders; and subsequent feeding difficulties and speech disorders in early childhood, dental anomalies, and communication ability. The secondary objective was to determine whether the SATB2 phenotype, when associated with an orofacial anomaly (BU, CP, or RS), is more severe for feeding and communication aspects than the SATB2 phenotype without orofacial anatomical anomaly. We finally looked for associations between oral phenotype and genotype.

## Material and methods

### Patients

All patients known to university hospital genetics departments in France, regardless of age, who carried a pathogenic or possibly pathogenic alteration of the *SATB2* gene were sought from large national networks for rare diseases (CRMR-SPRATON, AnDDiRares, and the FeCLAD network of clinical genetics laboratories) and the association of parents of children with SAS.

Different techniques were used for the genetic diagnosis: high throughput sequencing on targeted panels, whole exome sequencing, CGH array, and SANGER sequencing of *SATB2*. The type of molecular alteration was collected. The molecular abnormalities were divided into 3 groups: one with deletions that included *SATB2*, the second with mutations leading to a frameshift and/or a codon stop, and the third with missense mutations producing a defective protein.

The families were contacted by mail to ask them to participate in the study.

### Study of the clinical phenotype

Clinical information was collected from a questionnaire created for the study (Appendix 1), completed by the referring geneticists or pediatricians. These data were then completed by the parents in a telephone interview with NV. The information comprised perinatal events, first symptoms, early sucking capacity and early signs of velopharyngeal insufficiency, feeding problems to the age of 3 years, and early communication (pre-language from birth to 18 months) and then in early childhood (from 18 months to 6 years). The characteristics of communication in infancy were defined based on the Alarm Distress Baby Scale from 0 to 18 months and in early childhood on the Checklist for Autism in Toddlers scale from 18 months to 6 years [25].

The clinical data included orofacial and dental morphology and the existence of other malformations. The family’s socio-occupational category was noted and classified according to the French *Institut National de la Statisiques des Etudes Economiques* scale.

To look for associations between phenotype and genotype, we classified the severity of the orofacial anomaly in three groups (0: absence of anomaly; 1: RS spectrum [BU, CP or RS]); severity of feeding difficulties in two groups (0: no trouble; 1: swallowing disorders); severity of language deficit in three groups (0: normal language or simple sentence; 1: combine words or 10 meaningful words; 2: no language); and severity of communication behavior in three groups (0: normal; 1: agitation; 2: autistic features).

### Statistical analysis

Categorical variables are described with number (percentage) and quantitative variables with median (range). Patients were divided into two groups by the presence or absence of orofacial anatomical anomalies. The three anatomical abnormalities (BU, CP, and RS) were grouped together because they are part of the same spectrum and because the first analysis of these items separately in each group showed that results were comparable between them. Clinical data on feeding, communication, and speech capacities were compared between the two groups by Fisher’s exact test. A Fisher test was used to determine the association between mutation types and the severity of anatomical, feeding, language, and behavioral disorders. Statistical analyses were performed with GraphPad Prism 7.04.

### Ethics

The Necker Hospital ethics committee approved this study (Ethics committee registration no.: NCK-2020-E-006 TH VEGAS SATB2). After written and oral information was given, all parents consented in writing to the collection of data from the referring physicians and to their own oral interviews.

## Results

**Fig. 1:**
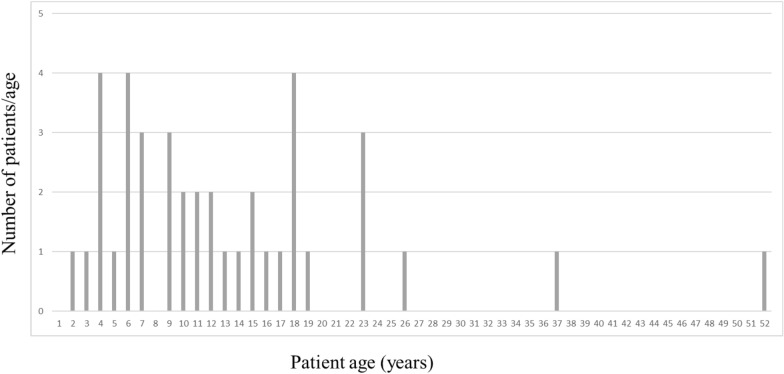
Bar chart showing the distribution of ages of the patients in our series

Of the 48 files reported by genetics teams, we included 40 patients, for a response rate of 83% of the families; 38 parents were able to be contacted by telephone. These 40 patients (23 boys) were from age 2 to 52 years at inclusion (median = 11 years; mean = 13.2 years; SD = 9.77 years) (Fig. 1). Genetic results were available for 31 patients, finding 15 frameshift/codon-stop, 9 missense and 7 deletion mutations.

### Oral clinical phenotype of the 40 patients

Abnormalities were discovered antenatally in only 5 patients: one with retrognathia, one with a single umbilical artery, one with hydramnios, and two with fetal growth restriction. At birth, patients’ height and weight measurements were similar to those of the general population. The neonatal period was marked by feeding difficulties in 22/40 patients (55%). A sucking-swallowing disorder was noted in 20/39 (51%) patients, with impossible breastfeeding and very slow bottle-feeding in most of the patients: 17/19 (89.5%) and 15/19 (79%), respectively. Feeding by a nasogastric tube was necessary from the first days of life for 8 patients (8/40; 20%), for a mean of 10 weeks (8 days to 4 months). Nasal congestion was present in half the patients (20/40; 50%), with chronic pharyngeal itching in 4/36 (11.1%). Only one patient presented laryngeal stridor (1/38; 2.6%). Delay in chewing solid food was noted in half the cases (18/36; 50%) and aspiration in nearly one quarter (9/39; 23%).

In this cohort, an orofacial morphology anomaly was found in half the cases (20/40; 50%): 10/40 (25%) with RS, 8/40 (20%) with posterior CP, and 2/40 (5%) with only BU. Delayed eruption of the first tooth was common (19/23; 82%) at a median age of 12 months (range: 5–18 months) (Table 1).

**Table 1:** Clinical characteristics of the patients with SAS (n = 40)

Clinical characteristics	Patients	
	n	%
Sex male/female	23/17	57.5/42.5
*Prenatal and neonatal period*		
Prenatal ultrasound abnormalities	5/38	13
Gestational age at birth (median)	39 weeks	
Measurements at birth		
Weight < -2 SD	4	10
Height < -2 SD	3	7.5
Head circumference < -2 SD	0	0
*Orofacial anomalies*		
Bifid uvula	2	5
Posterior cleft palate	8	20
Robin sequence	10	25
*Feeding and velopharyngeal sufficiency (0–3 years)*		
Early feeding difficulties	22	55
Sucking-swallowing disorders	20/39	51.3
Nasal discharges	20	50
Stertor	4/36	11.1
Laryngeal stridor	1/38	2.6
Breastfeeding possible	8	20
Time to drink bottle > 30 min	15/34	44.1
Nasograstric tube feeding	8	20
Delay in chewing solids	18/36	50
Dysphagia aspiration	9/39	23
*Communication from birth to 18 months*		
Eye contact	32	80
Smile response at normal age	36	90
Preverbal communication	26	65
Absence of babbling	33/39	84.6
Limited interest in communication partners	15/39	38.4
Repetitive behaviors	6/39	15.4
Tactile irritability	5	12.5
Auditory hypersensitivity	8	20
*Communication and speech from 18 months to 6 years*		
Normal children’s group games	15/37	40.5
Eye contact and social smile	35	88
Response/reaction to own first name	39	97.5
Points at objects and at people	31	77.5
Delayed preverbal communication/language	19/38	50
Major speech delay	40	100
Hyperactivity	11	28
Repetitive behaviors	16	40
Auditory and/or tactile hypersensitivity	11/39	28
*Current speech level*		
Sentences	4	10
Combinations of words	8	20
Meaningful words (10 or more)	14	35
Sign language	25	62.5
Support (pictogram, tablet, Makaton)	26	65
*Dental abnormalities*		
Median age at eruption of first tooth, in months/n	12	19
Delay in loss of baby teeth	11/36	30
Macrodontia	24/36	67
Tooth malposition	18/36	50
Clinical absence of teeth	7/36	19
Abnormal tooth shapes	6/36	17
Number of patients	36	90
Gum hypertrophy	9/38	23.7
*Sleep and behavior disorders*		
Autistic traits	20	50
Sleep disorders	17	43

The first worries about development took place at a median age of 12 months (range: 2–36 months), with delays in psychomotor development (23/33; 70%) and speech (20/33; 61%) as the principal alerts. Looking back, parents reported that communication with their baby was abnormal from the first months of life, with poor babbling in 33/39 cases (85%). Eye contact and responsive smiles were mostly present, in 32/40 (80%) and 36/40 (90%) of patients, respectively. Limited interest in communication partners was noted in 15/39 children (38%), repetitive gestures in 6/39 (15%), auditory hypersensitivity in 8/40 (20%), and tactile irritability in 5/40 (12.5%).

From age 18 months to 6 years, eye contact and social smiles mostly continued (35/40; 88%), but normal ability for children’s group games was noted in only 15/37 (40.5%) patients.

A major speech delay (no more than 10 words at 3 years) was described in all patients, associated with delay in preverbal communication in half (19/38; 50%). Hyperactivity was noted in 11/40 patients (28%), repetitive behaviors in 16/40 (40%), and auditory and/or tactile hypersensitivity in 11/39 (28%).

At the last evaluation, at a median age of 9.5 years (range: 2–50 years), feeding difficulties persisted in more than half the patients (22/40; 55%), with drooling, chewing disorders that could lead to dysphagia aspiration or a gag reflex exacerbated by solid foods. Dental anomalies were described in most patients (36/40; 90%), with macrodontia (24/36; 67%), dental malpositions (18/36; 50%), clinical absence of teeth (7/36; 19%), and abnormally shaped teeth (6/36; 17%). Gum hypertrophy was found in 9/38 cases (24%). At the study time, severe speech delay remained in all patients. Only 4 patients were able to utter simple sentences (4/40; 10%) and only 8 patients could combine words (8/40; 20%). Isolated meaningful words could be used by 24 patients (24/40; 60%); the number ranged from 5 to 100 words per patient. Nonetheless, most had a vocabulary of no more than 20 words (14/18; 78%). Twenty-five patients had learned a sign language (25/40; 62.5%) that could range from 3 to 50 signs; 26 patients also used support such as pictograms, tablets or Makaton (26/40; 65%). Four children were able to read words (4/40; 10%). One patient had reportedly acquired limited writing skills (1/40; 2.5%).

Variable autistic traits were described in 20/40 patients (50%), including a limited interest in others, repetitive behaviors, limited interests, inability to tolerate frustration or change, or actual autism spectrum disorder.

### Feeding, communication, and speech in the presence of RS, CP, or BU (Table 2)

**Table 2:** Feeding, communication, and speech with or without orofacial anomaly

	Cleft+ group		Cleft− group		*P* value
Number of cases	20/40	50%	20/40	50%	0.3809
Median age at inclusion (in years)	9		13		
*Early feeding disorders*					
Feeding difficulties	19/20	95%	3/20	15%	< 0.0001
Sucking-swallowing disorders	18/19	94.7%	2/19	10.5%	< 0.0001
Stertor	4/16	25%	0/20	0%	0.03
Laryngeal stridor	1/18	5.6%	0/20	0%	0.47
Breastfeeding	2/18	11%	5/20	25%	0.41
Time to drink bottle > 30 min	13/15	86.7%	2/19	10.5%	< 0.0001
Nasogastric tube	8/20	40%	0/20	0%	0.003
Delay in chewing solids	12/17	70.6%	6/19	31.6%	0.04
Dysphagia aspiration	5/20	25%	4/19	21%	1
*Communication from birth to 18 months*					
Eye contact	13/20	65%	19/20	95%	0.0436
Smile response	18/20	90%	18/20	90%	1
Preverbal communication	10/20	50%	16/20	80%	0.0958
Absence of babbling	19/20	95%	14/19	73.6%	0.0915
Limited interest in communication partners	10/20	50%	5/19	26.3%	0.1908
Repetitive behaviors	2/20	10%	4/19	21%	0.4075
Tactile irritability	3/20	15%	2/20	10%	1
Auditory hypersensitivity	3/20	15%	5/20	25%	0.6948
*Communication speech and language from 18 months to 6 years*					
Normal children’s group games	7/19	36.8%	8/18	44.4%	0.7431
Eye contact and social smile	16/20	80%	19/20	95%	0.3416
Response/reaction to own first name	20/20	100%	19/20	95%	1
Points at objects and at people	15/20	75%	16/20	80%	1
Delayed preverbal communication/language	10/18	55.6%	9/20	45%	0.7459
Major speech delay	20/20	100%	20/20	100%	1
Hyperactivity	3/20	15%	8/20	40%	0.1552
Repetitive behaviors	8/20	40%	8/20	40%	1
Auditory and/or tactile hypersensivity	4/20	20%	7/19	36.8%	0.3008
*Current speech level*					
Sentences	0/20	0%	4/20	20%	0.106
Combinations of words	0/20	0%	8/20	40%	0.0033
Meaningful words (≥ 10 words)	2/20	10%	12/20	60%	0.0022
Sign language	11/20	55%	15/20	75%	0.3203
Support (pictogram, tablet, Makaton)	13/19	68.4%	12/20	60%	0.7411
Autistic traits	11/20	55%	9/20	45%	0.7524
*Dental anomalies*					
Dental anomaly	20/20	100%	16/20	80%	0.106
Macrodontia	14/20	70%	10/16	62.5%	0.7295
Tooth malpositions	8/20	40%	10/16	62.5%	0.3145
Clinical absence of teeth	5/20	25%	2/16	10%	0.4264
Abnormal tooth shapes	5/20	25%	1/16	5%	0.1962
Gum hypertrophy	8/20	40%	1/16	5%	0.1818
Tooth decay	5/20	25%	7/16	43.7%	0.2983
*Mutations*					
Frameshift/codon stop	9/17	52.9%	5/13	38.4%	0.4837
Missense	4/17	23.5%	5/13	38.4%	0.4434
Deletion	4/18	22.2%	3/13	23%	1

The group of patients presenting RS, CP or BU accounted for half of the total sample, with median age 9 years (range: 3–52). The CPs were all posterior median, with no lip or maxillary clefts. We called this group the Cleft**+ **group. The group with no orofacial anomaly (Cleft**−** group) comprised 20 patients with comparable median age of 13 years (range: 4–37 years). The families’ socioeconomic status was similar in the two groups. These similarities in age and socioeconomic status allowed for incriminating their orofacial anomaly in their language outcome.

Feeding difficulties were significantly more substantial in the Cleft+ than Cleft− group (*P* < 0.0001); that is, they were nearly constant in this group (19/20, 95%) but were present in less than one-quarter of the Cleft− group (3/20, 15%). In the Cleft+ group, both a sucking-swallowing disorder and spending more than > 30 min finishing a bottle were common, in 18/19 (95%) and 13/15 (87%) patients, respectively; a nasogastric tube had been necessary in 8/20 patients (40%). In the Cleft− group, only two patients had either of these disorders (2/19; 10.5%), and no patient required a nasogastric tube. In the Cleft+ group, signs of velopharyngeal insufficiency were logically constant; more rarely, stertor or stridor was found, in 4/16 (25%) and 1/18 patients (5.5%), respectively. A delay in chewing was observed in most patients (12/17; 71%) and dysphagia with aspiration of fluids or solids in one quarter (5/20; 25%). The Cleft− group showed no signs of velopharyngeal insufficiency or stertor or stridor. A delay in chewing was found in only one third of the patients (6/19; 32%) and early dysphagia aspiration in less than one quarter (4/19; 21%). Dental anomalies were systematic in the Cleft+ group but absent in 4 patients in the Cleft− group (*p* = 0.106). However, the two groups did not differ in the various possible dental anomalies (macrodontia malposition, clinical absence of teeth, abnormal tooth shape, gum hypertrophy, tooth decay).

The two groups did not differ in early communication, except for eye contact from birth to 18 months, which was normally present more often in the Cleft− than Cleft+ group. Autism spectrum disorders were found in fewer than half the cases, with limited interest in communication partners, repetitive behaviors, tactile irritability, and auditory hypersensitivity. The two groups did not differ significantly in these autistic traits.

From age 18 months to 6 years, progress in communication and speech was worse in the Cleft+ than Cleft− group. Despite major speech delay found in both groups, speech levels at the time of the study were significantly lower in the Cleft+ than Cleft− group; 40% of patients in the Cleft− group and none in the Cleft+ group could combine words together. A vocabulary of at least 10 meaningful words was acquired by 12/20 patients (60%) in the Cleft− group versus 2/20 (20%) in the Cleft+ group. Results were similar in the two groups for communication, eye contact, social smiling, preverbal language delay, and autistic traits.

### Genotype–phenotype association

To assess genotype–phenotype associations in SAS syndrome, we investigated the association between the severity score for selected criteria (anatomical, feeding, communication and behavior) and three genetic subgroups (frameshift/codon stop, missense, and deletion mutations). We found a significant association between type of genetic anomaly and severity of language impairment (*p* = 0.0088), with 86% in frameshift/codon stop, 72% in deletion mutations and 33% in missense mutations. No other difference appeared between the 3 groups of mutations (Table 3).

**Table 3:** Association between Phenotype and the 3 types of mutations

	Frameshift/stop codon		Missense		Deletion		*P* value
Number of cases	15	37.50%	9	22.5%	7	17.50%	
*Anatomical severity score*							0.716
(0) no anomaly	6/15	40%	5/9	56%	4/7	57%	
(1) RS spectrum	9/15	60%	4/9	44%	3/7	43%	
*Language severity score*							0.0088
(0) normal language or simple sentence	1/15	7%	0/9	0%	1/7	14%	
(1) word association or > 10 meaningful words	1/15	7%	6/9	67%	1/7	14%	
(2) no language	13/15	87%	3/9	33%	5/7	72%	
*Eating disorder severity score*							0.280
(0) no trouble	13/15	87%	6/9	67%	4/7	57%	
(1) swallowing disorder	2/15	13%	3/9	33%	3/7	43%	
*Comportemental severity score*							0.306
(0) normal	9/15	60%	2/9	21.5%	2/7	29%	
(1) agitation	1/15	7%	2/9	21.5%	2/7	29%	
(2) autistic trait	5/15	33%	5/9	55%	3/7	43%	

## Discussion

This work describes for the first time the oral phenotype, in terms of malformations and function, of a large series of patients with SAS in France. This series of 40 patients is important insofar as the US SATB2 registry currently includes 72 patients [26] in a country with a population of about 331 million inhabitants in comparison to the French population of 68 million.

We confirmed the severity of SAS on speech, with a complete absence of oral speech or a vocabulary of no more than 10 words in two-thirds of our cohort. These results are similar to those by Zarate et al., who found that 82% of the participants had a vocabulary of fewer than 10 words or no speech at all at age 10 years [26]. Also, we confirm that these patients—two-thirds of our cohort and one-third of the US cohort—use nonverbal communication with a gestural language (sign language, Makaton) or communication that uses visual supports (pictograms, tablet) [8, 27]. These results support that appropriate management of the language delay in patients with SAS requires the early establishment of nonverbal communication and speech therapy with diverse supports.

The first notable sign in terms of preverbal communication in the first months of life was the absence of babbling by a baby often described by parents as very calm. We found that language impairment was more severe in cases of frameshift/codon stop or deletion mutations. Indeed, more severe damage is expected in the absence of a protein or a truncated protein with reduced or no activity. Zarate et al. studied the association between phenotype and genotype of 158 individuals and found that the proportion of individuals with no language was lowest for those with nonsense variants and highest for those with missense mutations [28]. We cannot compare our results to those of Zarate et al. because our samples were very small, with only 31 known mutations (vs 158), and the mutations compared were not the same (missense, insertion, frameshift/codon stop, splice sit variant, deletion, duplication, and translocation in Zarate et al.). Half of our SAS children presented behavior disorders, especially autistic traits, which is comparable to Zarate et al. (56%) [27].

Several studies have shown the role of SATB2 in brain development, with expression in the cerebral cortex, cerebellum, hippocampus, and basal ganglia [29–32]. However, its real function remains unknown, and the pathophysiology involved in the neurological problems related to mutations or deletions of the *SATB2* gene is not yet understood. SATB2 is also described as expressed in the parabrachial nucleus, itself involved in the sensory processes of taste, pain, and thermoregulation [33]. Indeed, several parents have reported eating disorders in older children associated with a lack of satiety, compulsive eating, and hyposensitivity to pain. These aspects could not be quantified and would be interesting to study in more detail in future studies.

Our study also confirmed the high frequency of early feeding difficulties in children with SAS [26], with persistent drooling and chewing disorders in half the cohort. Hypotonia of the tongue and jaw was reported by some parents, but we have not yet been able to document this adequately in patients. This clinical sign would be interesting to quantify in future studies to determine its involvement in feeding difficulties and for adapting speech therapy.

Half of our participants had RS spectrum, BU, posterior CP, or complete RS. These figures are slightly higher than those reported in the US registry—a cohort of 72 individuals, 40% with CP and 45% with micrognathia [26]. A literature study of 40 patients [34] reported 47.5% with CP. Both studies did not specifically mention RS, but these patients accounted for 25% of our series. This difference is probably related to France having a specific rare-disease referral hospital for patients with RS and our testing for SATB2-related anomalies in children with RS for several years. Including RS as a specific sign in SAS seems important, especially for obstetricians and neonatologists. Therefore, anomalies of the *SATB2* gene must be included in the molecular abnormalities searched for prenatally when fetal retrognathia or RS is identified and in newborns under management for RS.

Our work showed that children with SAS including RS spectrum had more severe feeding and communication/speech phenotypes than those without these orofacial abnormalities. The greater severity of neonatal sucking-swallowing and feeding disorders was expected in this group, but the more severe involvement of speech and language was an unexpected finding. At the age when the children in the Cleft+ group are evaluated (9 years), a phonatory disorder related to a CP that has been surgically repaired can no longer be blamed for the speech delay. Neither the children’s age nor their parents’ socioeconomic category explains this difference between the two groups. Of note, Snijders Blok et al. reported more CPs in nonverbal individuals than in those with primary verbal communication [35].

One of the hypotheses explaining this difference in severity, could be that CP and RS are the scars of embryonic damage affecting both mandibular osteogenesis and migration of neural crests into the pharyngeal arches. As they grow up, individuals in the Cleft+ group could be investigated for bone fragility.

In mice, *Satb2* is expressed as early as E10.5 in the frontonasal processes and in the maxillary bud of the first pharyngeal arch [14]. Thus *Satb2*^*−/−*^ mice have a CP, a smaller lower jaw with an anterior defect, hypoplasia of the premaxilla and the nasocapsular region, and microcephaly [20, 36]. Studies of *Satb2*^*−/−*^ mouse embryos up to E14.5 suggest that the absence of palate closure results from a failure of fusion on the midline of the palatal processes and glossoptosis [20]. They also demonstrate increased apoptosis in the craniofacial mesenchyme and decreased expression of three genes: *Pax9,* involved in the development of the mandibular incisors, and *Msx1* and *Alx4,* involved in development of the palate [36]. Aside from the small sample size, we found no explanation for this difference in phenotype in the type of molecular anomaly. Liu et al. [34] analyzed the clinical manifestations and genetic variations of 39 previously published cases and observed that no hotspot mutation was found in cases of CP and the same mutation could be found with or without CP. This finding points to probable multifactorial causes in the development of CP in SAS syndrome.

To complete the orofacial phenotype, we also collected dental clinical phenotypes, and as in several previous studies, similar dental damage was found in most SAS patients [6, 8, 9, 27, 34, 37–40]. Scott et al. [40] described a unique and consistent clinico-radiological phenotype that should help in late diagnosis.

The strengths of this study are that it is based on national cohort, with data collected both from physicians referring patients but also directly from the parents, who are best able to inform about the first months of life of their child in terms of feeding and development. Its limitations are that it is based on a questionnaire about past clinical signs, relying on parents’ memories and thus creating variability in the quality of the data collection. Moreover, we did not use validated scales for testing the individuals. Finally, since this study was conducted by paediatricians, precisions about dental anomalies are limited.

In conclusion, this cross-sectional study confirms the severity of the early oral phenotype of SAS in terms of feeding, communication, and speech. It shows the more serious involvement of this oral phenotype in the presence of the RS spectrum phenotype. These points allow us to recommend that pediatricians closely monitor preverbal communication and psychomotor development in infants with apparently isolated BU, CP or RS and remain vigilant in the absence of babbling, to set up early psychomotor and speech therapy without waiting for the absence of language at age 3 years. Anomalies of *SATB2* should also be part of the exploratory genetic work-up for apparently isolated CP or RS, especially in the prenatal period.

## Data Availability

The datasets used and/or analysed during the current study are available from the corresponding author on reasonable request.

## Abbreviations

CP: Cleft palate
BU: Bifid uvula
FGR: Fetal growth restriction
NGT: Nasogastric tube
RS: Robin sequence
SAS: SATB2 associated syndrome
